# A Convenient Cas9-based Conditional Knockout Strategy for Simultaneously Targeting Multiple Genes in Mouse

**DOI:** 10.1038/s41598-017-00654-2

**Published:** 2017-03-31

**Authors:** Jiang Chen, Yinan Du, Xueyan He, Xingxu Huang, Yun S. Shi

**Affiliations:** 1 State Key Laboratory of Pharmaceutical Biotechnology and MOE Key Laboratory of Model Animal for Disease Study, Model Animal Research Center of Nanjing University, Nanjing, 210061 China; 2 School of Life Science and Technology, Shanghai Tech University, 100 Haike Rd., Pudong New Area, Shanghai, 201210 China; 3 Department of Microbiology and Parasitology, Anhui Medical University, Hefei, 230032 China

## Abstract

The most powerful way to probe protein function is to characterize the consequence of its deletion. Compared to conventional gene knockout (KO), conditional knockout (cKO) provides an advanced gene targeting strategy with which gene deletion can be performed in a spatially and temporally restricted manner. However, for most species that are amphiploid, the widely used Cre-flox conditional KO (cKO) system would need targeting loci in both alleles to be loxP flanked, which in practice, requires time and labor consuming breeding. This is considerably significant when one is dealing with multiple genes. CRISPR/Cas9 genome modulation system is advantaged in its capability in targeting multiple sites simultaneously. Here we propose a strategy that could achieve conditional KO of multiple genes in mouse with Cre recombinase dependent Cas9 expression. By transgenic construction of loxP-stop-loxP (LSL) controlled Cas9 (LSL-Cas9) together with sgRNAs targeting EGFP, we showed that the fluorescence molecule could be eliminated in a Cre-dependent manner. We further verified the efficacy of this novel strategy to target multiple sites by deleting *c*-*Maf* and *MafB* simultaneously in macrophages specifically. Compared to the traditional Cre-flox cKO strategy, this sgRNAs-LSL-Cas9 cKO system is simpler and faster, and would make conditional manipulation of multiple genes feasible.

## Introduction

In biomedical studies, one of the most efficient methods in understanding gene function is to delete it and examine the resulting physiological changes. The removal of the DNA sequence in embryonic stem (ES) cells allowed the production of mice with specific gene knockout (KO)^1^. This approach has greatly advanced biomedical studies but suffers from some limitations. First, a significant subset of genes is essential for survival and genomic deletion in animals is lethal. Second, gene deletion in germline often leads to developmental compensation. These problems have been circumvented by generating conditional knockout (cKO) animals, in which the target gene is flanked with loxP sites^2^. The gene deletion can be precisely controlled by spatio-temporally defined expression of Cre-recombinase^3^. This method is powerful and widely used in biomedical studies. However, the method is time and labor consuming, and can be improved. First, it requires the generation of mice with genetic modification at precise positions. Second, for most of the mammalian genes that have two copies, additional breeding is needed to generate homozygous animals with both alleles loxP flanked (flox). Third, if one wish to delete several genes^4, 5^, multiple rounds of crossbreeding will have to be carried out and thus the labor would be exponentially increased^6^. Furthermore, this strategy is potentially restricted for multiple flox loci because of possible inter-locus recombination.

The clustered regularly interspaced short palindromic repeats (CRISPR)/CRISPR associated protein 9 (Cas9) system is recently used as a powerful genome-engineering tool in many species^7^. CRISPR/Cas9-mediated genome editing requires the endonuclease Cas9 and a single guide-RNA (sgRNA) which is a fusion construct of two short RNAs, a target-recognizing CRISPR-RNA, and a Cas9-recruiting tracer-RNA. When co-expressed with an appropriate sgRNA, Cas9 is recruited to the genomic DNA in a sequence specific manner, and cuts both strands at a precise location^8–10^. The genomic DNA is then repaired by non-homologous end joining (NHEJ), introducing mutations that could effectively interrupt the open reading frame, and thereby results in a functional KO of the encoded protein. Compared to other genome editing methods, the CRISPR/Cas9 system has a significant unique advantage in that it can target multiple genomic sites simultaneously^9^. Therefore, in this study we sought to use loxP-stop-loxP (LSL) cassette to control the Cas9 expression to achieve efficient cKO. By targeting EGFP or *c*-*Maf/MafB* genes using specified sgRNAs, we prove the principle that one-step transgenic construction of LSL-Cas9 together with specified sgRNAs can serve as a fast and convenient cKO strategy.

## Results

### Generation of Cas9-based EGFP cKO mice

**Figure 1:**
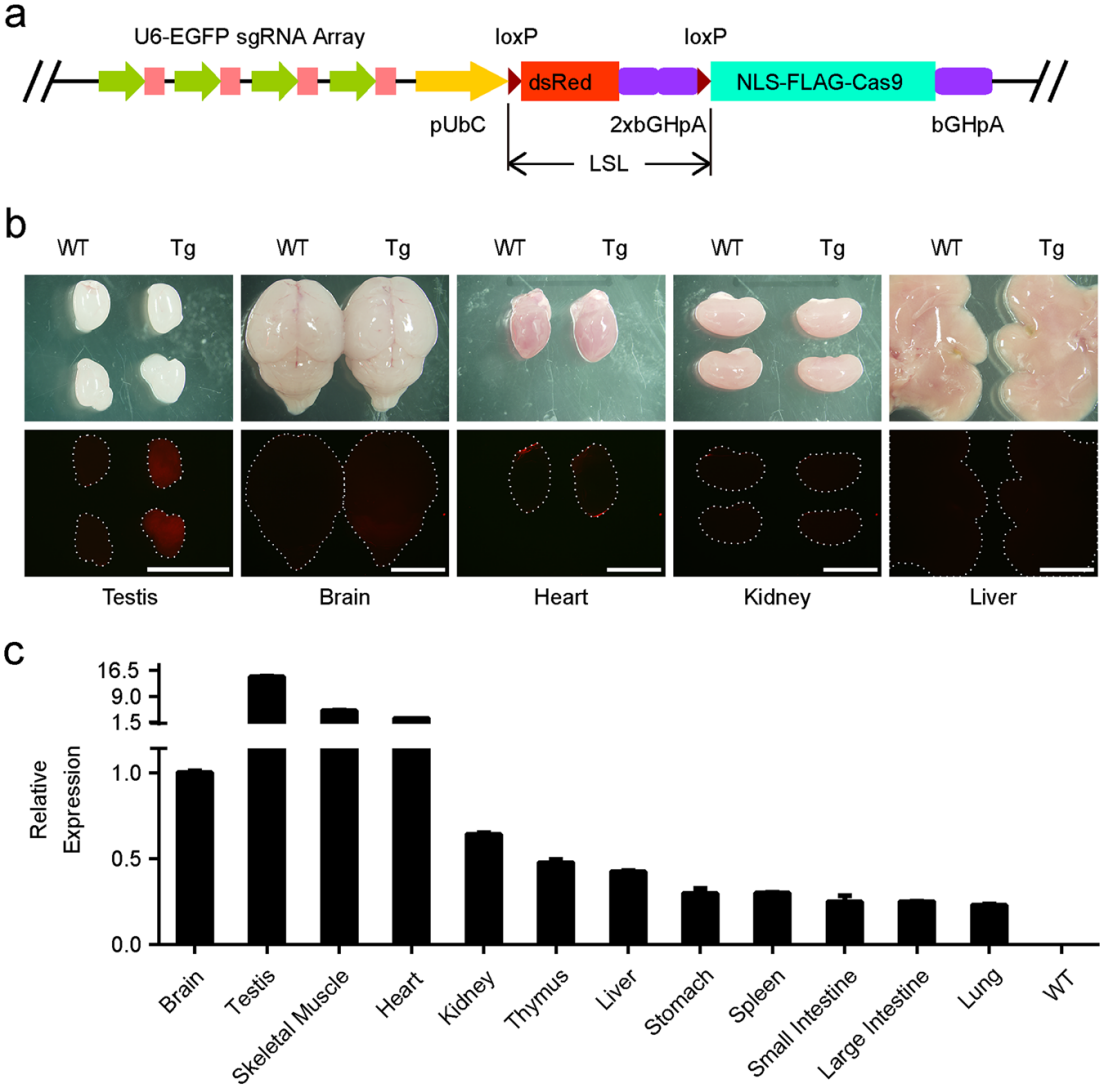
Generation of transgenic Cas9-based EGFP conditional KO mouse. (**a**) Schematic of sgRNAs^EGFP^-UbC-LSL-Cas9 transgenic vector. Four sgRNAs (pink) driven by U6 promoters (green arrowheads) were used to target independent sites of EGFP coding sequence. The Cas9 with nuclear localization signal (NLS) and FLAG tag was fused to LSL element and driven by UbC promoter. (**b**) DsRed fluorescence in organs of *sgRNAs* ^*EGFP*^-*LSL*-*Cas9* mouse (Tg) line 27. The upper panel shows organs in bright field. The lower panel shows dsRed fluorescence. Dotted lines indicate the organs invisible. Scale bar, 5 mm. (**c**) DsRed mRNA expression level in organs of mouse line 27 was analyzed by RT-PCR. The expression level of dsRed relative to β-actin in different organs was normalized to that in brain. WT mouse RNA mixture was absent of dsRed mRNA and used as a negative control (n = 3 repeats).

In order to test the idea that LSL controlled CRISPR/Cas9 can be used to achieve cKO in mice, we selected EGFP as target molecule and made transgenic mouse *sgRNAs* ^*EGFP*^-*LSL*-*Cas9*. The LSL element was the coding sequence for dsRed with an array of 2x BGH-PolyA surrounded by two loxP sites in the same direction. The dsRed was included with the intention to monitor the transcription of the transgene. The LSL-Cas9 was driven by ubiquitin C promoter (UbC). An array of 4 sgRNAs driven by U6 promoters was used to target EGFP coding sequence (Fig. 1a, Supplementary Table S1). This transgenic vector was then linearized and injected into the mouse zygotes. We obtained 38 founders, 9 of them were positive with transgenes. After mating with wild type (WT) C57BL/6JNju mice, three founders produced positive transgenic offspring steadily. We found the dsRed fluorescence were intensive in testis, but quite weak in other tissues (Fig. 1b), consistent with previous report that UbC promoter functions strongest in testis^11^. Since the UbC promoter is one of the widely used global promoters, we suspected the dsRed might be expressed at a low level in tissues other than testis. Therefore, we quantified and compared the transcription levels of dsRed mRNA in different tissues with real-time quantitative PCR (RT-PCR). In the line 27, we found that the highest expression was in testis, around 15 times of that in brain, consistent with the fluorescence observation. The mRNA in skeletal muscles and hearts was around 3–5 times of that in brain while it was lower in other tissues including lung, liver, spleen, kidney and digestion tracts (0.1–0.6 times of that in brain) (Fig. 1c). These results suggested that if Cas9 is induced by Cre-recombinase, its expression could be low in most tissues. The dsRed expression profile of the other two transgenic lines (lines 8 and 22) was similar (Supplementary Fig. S1a,b).

### EGFP targeting efficiency by Cre induced Cas9 in the transgenic mice

**Figure 2:**
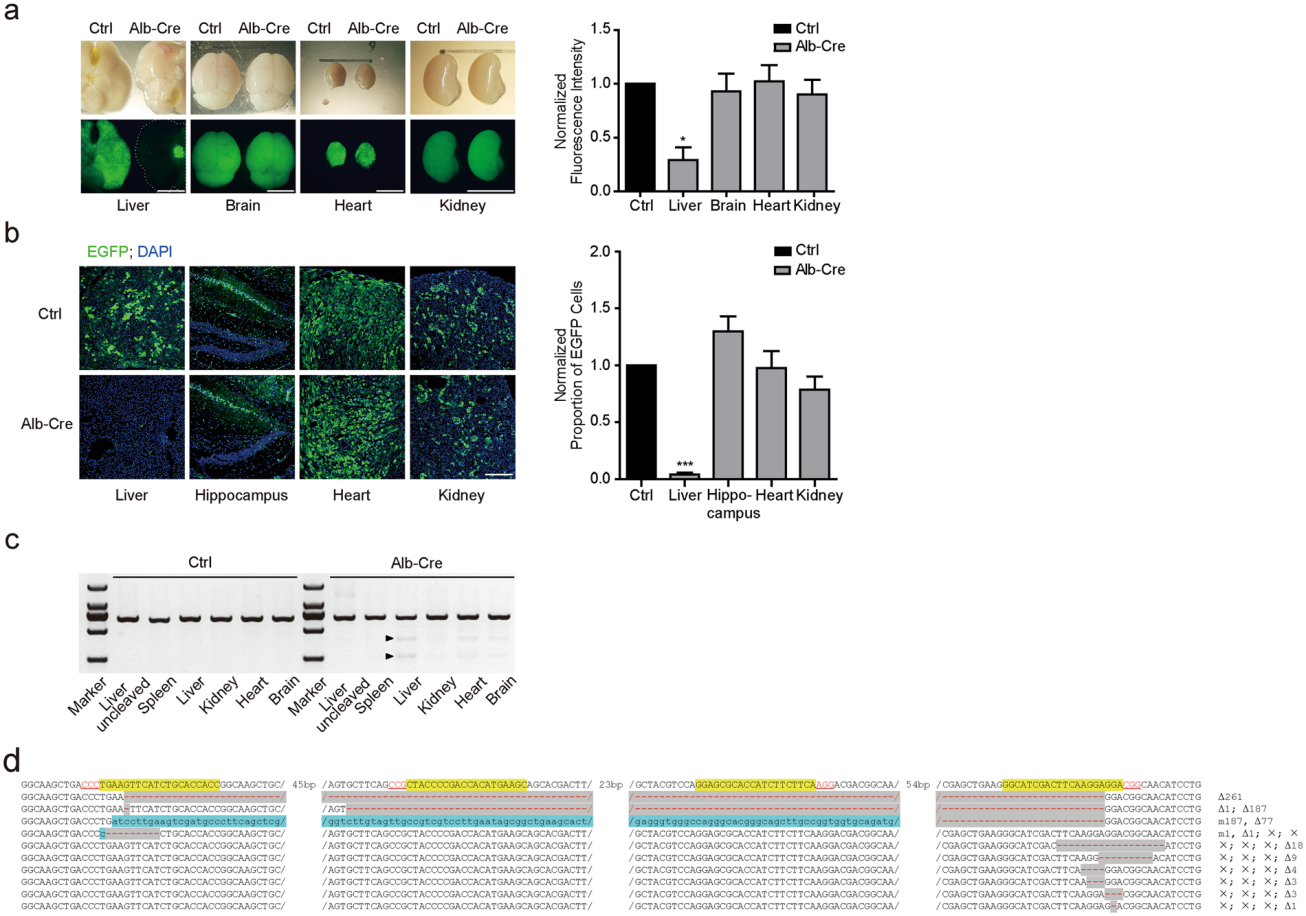
Tissue-specific mutations of EGFP in *sgRNAs* ^*EGFP*^-*LSL*-*Cas9*; *EGFP*; *Alb-Cre* mice. (**a**,**b**) EGFP fluorescence of liver was almost completely eliminated in *sgRNAs* ^*EGFP*^-*LSL*-*Cas9*; *EGFP*; *Alb*-*Cre* (Alb-Cre) mouse. Control (Ctrl) was *sgRNAs* ^*EGFP*^-*LSL*-*Cas9*; *EGFP* mouse. The right panels quantified fluorescence. (**a**) Paired t-test. liver, *p = 0.0109; brain, p = 0.8084, not significant (ns); heart, p = 0.8893, ns; kidney, p = 0.3101, ns. n = 3. (**b**) Paired t-test, liver, ***p = 0.0002; brain, p = 0.1486, ns; heart, p = 0.8784, ns; kidney, p = 0.2086, ns. n = 3. Scale bar, 5 mm for (**a**); 200 μm for (**b**). (**c**) T7EN1 assay showed obvious digested bands (arrows) indicating mutations in the lane of Alb-Cre positive mouse, while none in Cre negative mouse (Ctrl). M, maker. (**d**) Mutated EGFP sequences from *sgRNAs* ^*EGFP*^-*LSL*-*Cas9*; *EGFP*; *Alb*-*Cre* mouse liver. PAM (Red), sgRNAs targeting sites (yellow grounding), deletion (grey grounding) and base changing (blue grounding) are shown. Δ, deletion; m, mutation; ×, no mutation in the presenting sequence. Numbers following the symbols are base quantity of mutation.

To test whether the low expression of Cas9 is sufficient in incapacitating the target gene *in vivo*, we select liver, one of tissues that has the lowest expression, to analyze the efficacy of Cas9 mediated gene disruption. We crossbred the *sgRNAs* ^*EGFP*^-*LSL*-*Cas9* mice with a EGFP transgenic line, *CAG*-*EGFP*, which expresses EGFP globally^12^. As expected, almost all tissues showed green fluorescence (Fig. 2a, Ctrl). Then we crossbred the *sgRNAs* ^*EGFP*^-*LSL*-*Cas9*; *EGFP* animals with *Alb*-*Cre* line, a transgenic mouse line that Cre recombinase is specifically expressed in hepatic cells^13^. We found that the EGFP fluorescence in liver was almost disappeared, whereas in control tissues including heart, brain, and kidney, the EGFP signal was unchanged (Fig. 2a, *Alb*-*Cre*). Tissue sections showed the same results (Fig. 2b). The EGFP-positive cells in hippocampus, heart and kidney were not different in *Alb*-*Cre* positive and negative mice. In liver, the EGFP positive cells were rare in *Alb*-*Cre* positive animals (Fig. 2b). The residual of EGFP fluorescence shown in whole tissue liver and a few cells in tissue sections was probably due to the EGFP signal in non-hepatic cell types without Cre expression in liver or possible incomplete mutation by Cas9. T7EN1 (T7 endonuclease 1) assay^14^ of the genomic DNA from different tissues of the *sgRNAs* ^*EGFP*^-*LSL*-*Cas9*; *EGFP*; *Alb*-*Cre* mouse demonstrated the efficient mutation in EGFP locus in liver (Fig. 2c). T7EN1 digestion bands were also found in brain, heart and kidney, but were much weaker than in liver. To further confirm the mutations of EGFP in liver, we cloned the genomic EGFP coding sequences, inserted into pMD19-T vectors. We sequenced 23 clones, and 17 clones showed various mutations in EGFP segments (Fig. 2d, Supplementary Table S2), accounting for a mutation rate of 73.9%. Among these mutants, 10 had mutations at all four sgRNAs targeting sites. One had mutation at EGFP sgRNA1 site and the rest involved mutations at sgRNA4 site. The results clearly demonstrated that the Cre induced Cas9, whose expression level might be low, is sufficient to dysfunction target genes. In this study, Cre positive cells will constantly express DNA nuclease Cas9. In order to test whether the constant expression of Cas9 is harmful to the animal, we crossbred the *sgRNAs* ^*EGFP*^-*LSL*-*Cas9* mice with *DDX4*-*Cre* mouse line in which Cre recombinase is expressed in germline cells^15^ so that Cas9 should be globally expressed in the progenies. The progenies of *sgRNAs* ^*EGFP*^-*LSL*-*Cas9*; *DDX4*-*Cre* mice appeared normal (Supplementary Fig. S1c) and reproductive, consistent with a previous study^16^ demonstrating Cas9 is safe for animals.

### Efficient double cKO endogenous *c*-*Maf/MafB* genes by Cre induced Cas9 in the transgenic mice

**Figure 3:**
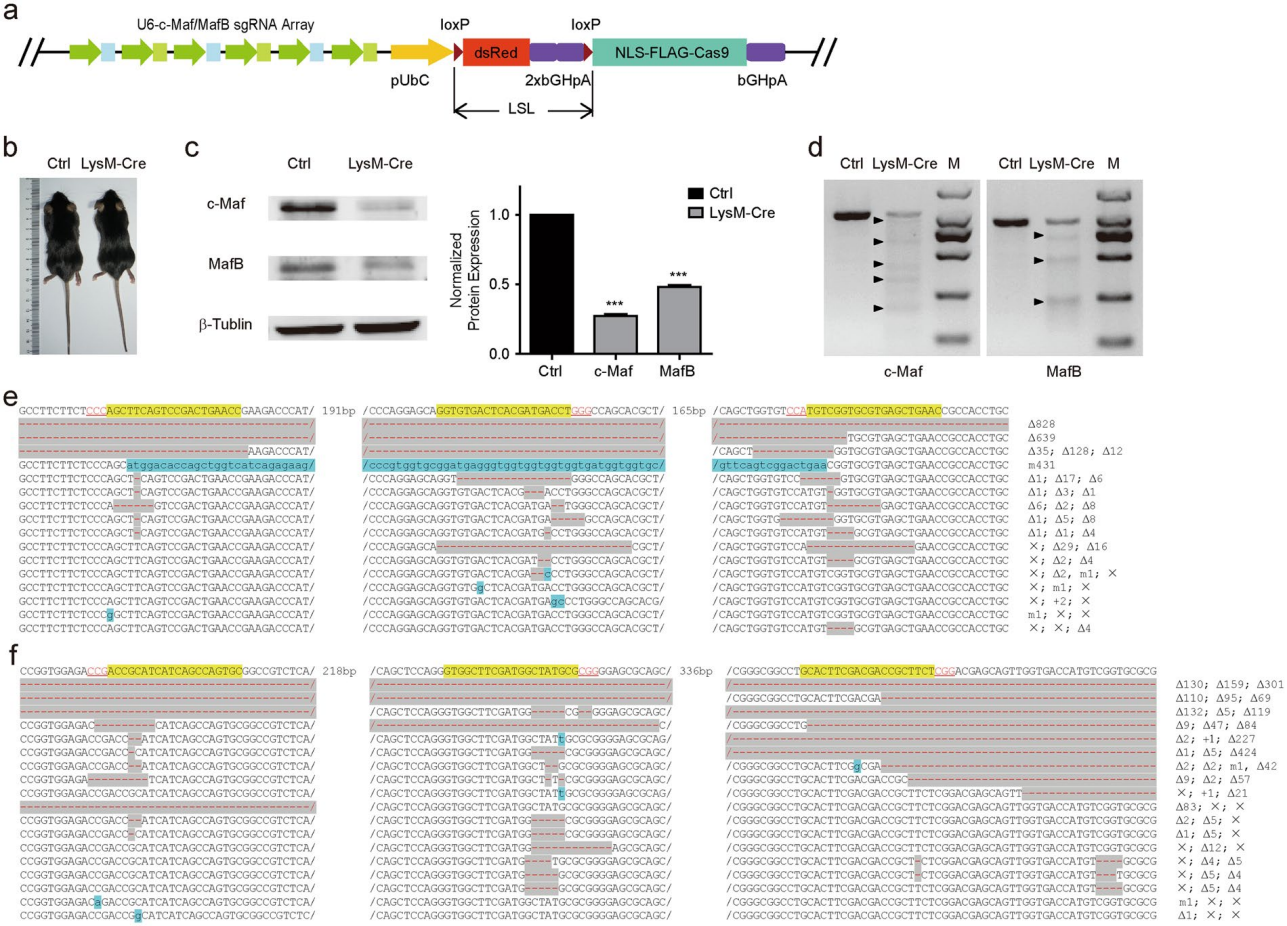
*c*-*Maf/MafB* double cKO via sgRNAs-LSL-Cas9 system. (**a**) Schematic of transgenic vector sgRNAs^*c*-*Maf/MafB*^-UbC-LSL-Cas9. Three U6 promoters (green arrowheads) driven *c*-*Maf* sgRNAs (blue blocks) and three U6 promoters driven *MafB* sgRNAs (green blocks) were arranged alternately. Other functional regions are combined as those in the sgRNAs^EGFP^-UbC-LSL-Cas9 transgenic vector (Fig. 1a). (**b**) Photograph of *sgRNAs* ^*c*-*Maf/MafB*^-*UbC*-*LSL*-*Cas9* mice. Ctrl, transgenic mouse without Cre. LysM-Cre, transgenic mouse express lysM-Cre. (**c**) Western blots of *c*-*Maf/MafB* double cKO macrophages (paired t-test, c-Maf, ***p = 0.003, n = 3. MafB, ***p = 0.0009, n = 3). (**d**) T7EN1 analysis of *c*-*Maf* and *MafB* mutations in the macrophages of a *sgRNAs* ^*c*-*Maf/MafB*^-*LSL*-*Cas9*; *LysM*-*Cre* (LysM-Cre) mouse. The control (Ctrl) was a *sgRNAs* ^*c*-*Maf/MafB*^-*LSL*-*Cas9* mouse without Cre recombinase. M, marker. (**e**) *c*-*Maf* mutation mode of the Cre-expressing macrophages of the transgenic mouse. (**f**) *MafB* mutation mode of the Cre-expressing macrophages of the transgenic mouse.+, insertion.

After verifying the targeting efficiency of EGFP by the novel cKO system, we wondered whether this approach could be used to cKO multiple endogenous genes. We chose to target *c*-*Maf* and *MafB* genes encoding transcription factors expressed in macrophages^17^. We designed three sgRNAs targeting each gene and arranged them alternately upstream to the LSL-Cas9 segment in the transgenic vector (Fig. 3a). Forty-two mouse founders were established and 4 of them were positive with the *sgRNAs* ^*c*-*Maf/MafB*^-*LSL*-*Cas9* segment in genome. Two lines (line 28 and 33) of them could produce transgenic offspring steadily. We crossbred line 33 with *LysM*-*Cre* mice, in which Cre recombinase is expressed in macrophages^18^. No obvious differences was seen between *c*-*Maf /MafB* double cKO mice and control mice without Cre (Fig. 3b). Western blotting of c-Maf and MafB from purified macrophages (Supplementary Fig. S2a) showed that the expression of both proteins decreased significantly (Fig. 3c). T7EN1 assay of macrophage genomic DNA from *sgRNAs* ^*c*-*Maf/MafB*^-*LSL*-*Cas9*; *LysM*-*Cre* mice showed obvious digested bands, indicating that *c*-*Maf* and *MafB* were mutated in macrophages of these animals (Fig. 3d and Supplementary Fig. S2b). Sequencing results of the clones of *c*-*Maf* or *MafB* gene segments confirmed that indels occurred at targeting loci (Fig. 3e,f). In 33 *c*-*Maf* clones that were sequenced (Supplementary Table S3), 19 were mutant resulting in a mutation rate of 57.6%. About 63% (12 of 19) of mutant *c*-*Maf* clones involved mutations at all three sgRNAs targeting sites, while those with mutations at two sites and single site were 10.5% and 26.3%, respectively. We sequenced 19 *MafB* clones and all had mutations, accounting for 100% mutation rate (Supplementary Table S4). The mutation rates at single, double and triple sites were 21.1%, 31.6% and 47.3% respectively. These results indicated that efficient cKO of genes could be approached with sgRNAs-LSL-Cas9 transgenic strategy.

## Discussion

In this study, we show that transgenic construction of LSL-Cas9 with specified sgRNAs could be used as a conditional knockout strategy. We would like, in theory, to compare this novel cKO system with the widely used Cre-flox cKO system. First, the LSL-Cas9 based cKO system only requires a one-step transgenic construction instead of germline modulation of genome at precise positions in the Cre-flox cKO system. Second, the traditional cKO approach is a *cis* system in which, deletion of the target gene requires both alleles to be loxP flanked. In practice, this need additional breeding and genotyping. The LSL-Cas9 based cKO system is a *trans* system, single copy of the transgenic construction is effective in deleting target gene, thus the laborious breeding and genotyping can be saved (See Supplementary Fig. S4 for a theoretical comparison between these two methods). This is very significant if multiple genes are to be deleted simultaneously. In the Cre-flox cKO system, it would take multiple rounds of breeding and genotyping to acquire the proper multiple flox mice. The workload would exponentially increase with the number of genes involved. Now, using the novel LSL-Cas9 strategy, there would still be a one-step transgenic by including more specified sgRNAs (Supplementary Fig. S4). In addition, in an extreme condition when the target genes are located in the same chromosome, the breeding strategy in Cre-flox cKO system would become much more difficult for that the positive rate of offspring would completely depend on the recombination rate between these genes^6^, thus often impracticable. This in LSL-Cas9 cKO system, however, would still be as simple as one-step transgenic. Furthermore, inter-locus recombination of multiple flox loci may occur in traditional cKO method while manipulating multiple genes. The LSL-Cas9 based strategy can circumvent this issue. The theoretic comparison between these two systems suggests the LSL-Cas9 cKO system is much simpler and more convenient.

One concern about this new cKO strategy would be that the Cre-recombinase releases the constant expression of Cas9. Is Cas9 harmful to animals? In our experience, we found the animals with global expression of Cas9 are rather normal without any observable health problems. The same observations were also made in recent Cas9 knockin animals^16^, suggesting the Cas9 is much safer than previously suspected. Another concern would be the off-target problems. So far the off-target has not been a huge problem for Cas9. Indeed, the off-target rate is rather lower than expected^19^. Besides, efforts were made to reduce the off-target rate^20–24^. In addition, although we did not observe the EGFP fluorescence change in tissues other than liver in *sgRNAs* ^*EGFP*^-*LSL*-*Cas9*; *EGFP*; *Alb*-*Cre* mice (Fig. 2a and b), we saw weak T7EN1 digestion bands in brain, heart and kidney (Fig. 2c). This is likely due to the Alb-Cre non-specific expression or the leaky of the Cas9 expression in those tissues. A stronger stop-signal in LSL cassette would reduce the leaky expression of the Cas9. Furthermore, we also noticed that different sgRNAs have variable efficacy. An optimized series of sgRNAs would improve the cKO efficiency.

In summary, we proved in principle a convenient conditional knockout strategy for simultaneously targeting multiple genes in mouse using sgRNAs-LSL-Cas9 cKO system.

## Methods

### Animals

Mice were housed in standard cages in an Assessment and Accreditation of Laboratory Animal Care–credited specific pathogen–free (SPF) animal facility on a 12 h light–dark cycle. All experiments involving use of mice were conducted in accordance with animal protocols approved by the Animal Care and Use Committee of the Model Animal Research Center, the host for the National Resource Center for Mutant Mice in China, Nanjing University. Both male and female animals were used in all the animal experiments.

### Vector Constructs

In order to generate transgenic mice, we constructed pU6-sgRNAs^EGFP^-UbC-LSL-Cas9 and pU6-sgRNAs^*c*-*Maf/MafB*^-UbC-LSL-Cas9 vectors. We used specified primers (Supplementary Table S5) and pUC57-sgRNA vector (Supplementary Table S6) as the template to PCR amplify DNA fragments with coding region of U6-sgRNAs for EGFP, *c*-*Maf* and *MafB*. After *BsmBI* digestion, the PCR products and pU6-*BsmBI*-UbC-LSL-Cas9 (Supplementary Table S6) were assembled by Golden Gate method to obtain pU6-sgRNAs^EGFP^-UbC-LSL-Cas9 and pU6-sgRNAs^*c*-*Maf/MafB*^-UbC-LSL-Cas9 vectors, which were linearized by *SalI* and purified by gel extraction for further mouse embryo injection.

### Embryo Injection

Mouse zygotes were obtained by mating of CBA males with superovulated C57BL/6J females. Linearized pU6-sgRNAs^EGFP^-UbC-LSL-Cas9 or pU6-sgRNAs^*c*-*Maf/MafB*^-UbC-LSL-Cas9 (4 ng/μl) were microinjected into the larger (male) pronucleus of fertilized oocytes. Injected zygotes were transferred into pseudopregnant female mice.

### Imaging

Postnatal day 20 (P20) mice were anesthetized with intraperitoneal injection of 1% pentobarbital sodium before cardiac perfused with phosphate-buffered saline (PBS) followed by 4% paraformaldehyde (PFA). Organs were fixed with PFA for less than 2 hours and photographed with a stereoscopic microscope for EGFP or dsRed fluorescence. Tissue sectioning were performed on a cryostat microtome at the thickness of 20 μm. The sections were transferred to glass slides and stained with DAPI (Sigma-Aldrich, D9542), and then sealed with cover slips. DAPI and EGFP fluorescence were observed using confocal microscopy.

### Real-time Quantitative PCR

RT-PCR was conducted to examine the differential expression of dsRed in the organs of *sgRNAs* ^*EGFP*^-*LSL*-*Cas9* mice. Total RNA was extracted from the organs of P30 mice with Trizol and isopropanol. For each organ, 1 μg total RNA was used to synthesize the first-strand cDNA using a reverse transcription kit (Vazyme, R123–01). The cDNAs were diluted in 10 times DEPC water and used as template for RT-PCR reaction (Vazyme, Q141-02). β-actin was used as an internal control. The primers for RT-PCR were: dsRed-forward (5′-GATCCACAAGGCCCTGAAGC-3′), dsRed-reverse (5′-GCTCCACGATGGTGTAGTCC-3′), β-actin-forward (5′-GGATGCAGAAGGAGATTACTG-3′) and β-actin-reverse (5′-CCGATCCACACAGAGTACTTG-3′).

### T7EN1 Assay and Sequencing

The T7EN1 cleavage assay was performed as previously described^14^. Briefly, the genomic DNA of samples (postnatal day 20) was purified and the target sequences were amplified using PCR. Purified PCR product went through denaturation and annealing process in a thermal cycler (Applied Biosystems) using the following protocol (95 °C, 5 min; 95–85 °C at −2 °C/s; 85–25 °C at −0.1 °C/s; hold at 4 °C). About 200 ng annealing product was digested with T7 endonuclease 1 (New England Biolabs) at 37 °C and analyzed by 3% agarose gel electrophoresis. The cleavage bands by T7EN1 cleavage assay indicated the modification of target loci. The PCR products were also subcloned into pMD19-T vector (TAKARA) for sequencing. The primers used to amplify EGFP, *c*-*Maf* and *MafB* are listed in Supplementary Table S7.

### Macrophage Induction

Isolation and culture of macrophages from 3–6 month old *sgRNAs* ^*c*-*Maf/MafB*^-*LSL*-*Cas9*; *LysM*-*Cre* and *sgRNAs* ^*c*-*Maf/MafB*^-*LSL*-*Cas9* mice were performed as previously reported^25^. Briefly, peritoneal exudate cells containing macrophages were obtained from mice 4 days after intraperitoneal injection of 2.5 ml of 4% wt/vol Thioglycolate Broth (Fluka) by flushing the peritoneum with 10 ml of ice-cold PBS. The cells were washed by centrifugation and then suspended in DMEM (Gibco) containing 10% FBS, 50 units/ml penicillin and 50 µg/ml streptomycin. The cells were plated in 9 cm culture plates (1 × 10^6^ cells per ml medium) and incubated at 37 °C, 5% CO_2_ incubator; non-adherent cells were removed by vigorous washing with PBS twice 2 hours after plating^25^. The remaining adherent cells was collected 24 hours later and subjected to flow-cytometry to measure purity of macrophages. Genomic DNA of collected cells was extracted for T7EN1 assay and sequencing. C-Maf and MafB proteins were measured with western blotting.

### Flow-cytometry

The macrophages were digested with 0.1% EDTA 24 hours after plating and washed by centrifugation with PBS at 400 g/5 min. The cells were suspended in 100 μl PBS and incubated with anti-F4/80 (Biolegend, 123122) and anti-CD11b antibody (Biolegend, 101228) on ice for 2 hours. After washed with cold PBS and suspended, the macrophages were applied for flow-cytometry at BD FACSCaliber flow cytometer.

### Western Blots

Acquired macrophages (10^6^ cells per sample) were lysed in with 50 μl buffer composing of NaCl (150 mM), Tris (50 mM, pH 7.4), 1% Nonidet P-40, 0.5% sodium deoxycholate, and complete Protease Inhibitor Mixture Tablets (Roche). Cell lysates were placed in ice for 30 min, and centrifuged at 17,000 × g, 4 °C for 30 min. After centrifugation, the supernatant was mixed with 4 × loading buffer. The samples were cooked at 95 °C for 20 min and subjected to 10% SDS/PAGE gels electrophoresis. Protein bands were transferred to PVDF membranes (EMD Millipore) at 75 V for 3 h. The membranes were blocked for 1 h at room temperature in NaCl (150 mM), Tris-HCl (10 mM, pH7.6), and 0.1% Tween 20 containing 5% nonfat milk and then probed with anti-c-Maf antibody (Santa Cruz, sc-7866) or anti-MafB (Abcam, ab66506). ECL substrate (Thermo Fisher Scientific Life Sciences) was used for protein detection.

## Electronic supplementary material

Supplementary Information
