# Supplementary material for: A Convenient Cas9-based Conditional Knockout Strategy for Simultaneously Targeting Multiple Genes in Mouse
Source: Sci Rep. 2017 Mar 31;7:517. doi: 10.1038/s41598-017-00654-2 (PMC5428785; doi:10.1038/s41598-017-00654-2)
Supplement: Supplementary file 1 — Supplementary Information [file 41598_2017_654_MOESM1_ESM.doc]

**Supplemental Information**

**A Convenient Cas9-based Conditional Knockout Strategy for Simultaneously Targeting Multiple Genes in Mouse**

**Jiang Chen, Yinan Du, Xueyan He, Xingxu Huang, Yun S. Shi**

**Supplemental Information Inventory:**

**Supplementary Fig. S1. DsRed mRNA expression in *sgRNAs^EGFP^-LSL-Cas9* mice line 8 and 22 and pictures of adult wildtype mouse and mouse expressing Cas9 globally at the same age.**

**Supplementary Fig. S2. Flow-cytometry and T7EN1 analysis of *c-Maf* and *MafB* mutations in macrophages of *sgRNAs^c-Maf/MafB^-LSL-Cas9;LysM-Cre* mice.**

**Supplementary Fig. S3. Theoretical comparison of crossbreeding labor to generate cKO mice via sgRNAs-LSL-Cas9 and Cre-loxP strategies.**

**Supplementary Table S1. Sequences of sgRNAs targeting EGFP, *c-Maf* and *MafB*.**

**Supplementary Table S2. Summary of EGFP sequences from the liver of *sgRNAs^EGFP^-LSL-Cas9;EGFP;Alb-Cre* mice.**

**Supplementary Table S3. Summary of *c-Maf* sequences from macrophages of *sgRNAs^c-Maf/MafB^-LSL-Cas9;LysM-Cre* mice.**

**Supplementary Table S4. Summary of *MafB* sequences from macrophages of *sgRNAs^c-Maf/MafB^-LSL-Cas9;LysM-Cre* mice.**

**Supplementary Table S5. Primers for PCR amplification of the coding regions of U6-sgRNAs for EGFP, *c-Maf* and *MafB* from pUC57-sgRNA template.**

**Supplementary Table S6. Sequences of pUC57-sgRNA and pU6-*BsmBI*-UbC-LSL-Cas9.**

**Supplementary Table S7. Primers of PCR for T7EN1 and sub-cloning of EGFP, *c-Maf* and *MafB* segments.**


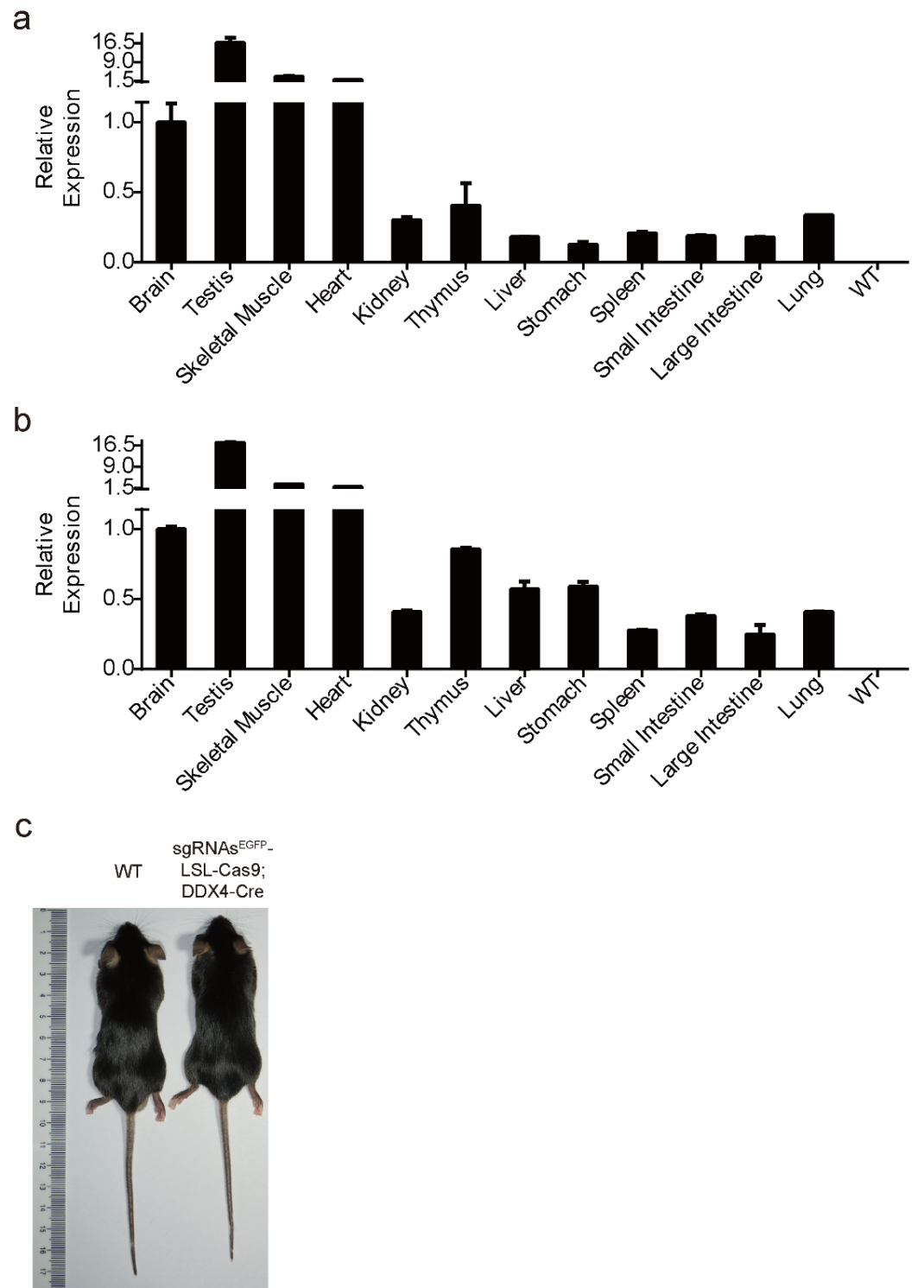


**Supplementary Fig. S1. DsRed mRNA expression in *sgRNAs^EGFP^-LSL-Cas9* mice line 8 and 22 and pictures of adult wildtype mouse and mouse expressing Cas9 globally at the same age.** (a, b) DsRed expression relative to β-actin in different organs were normalized to that in brain. (a) Line 8. (b) Line 22**.** (c) Pictures of adult wildtype (WT) and mouse expressing Cas9 globally.

**
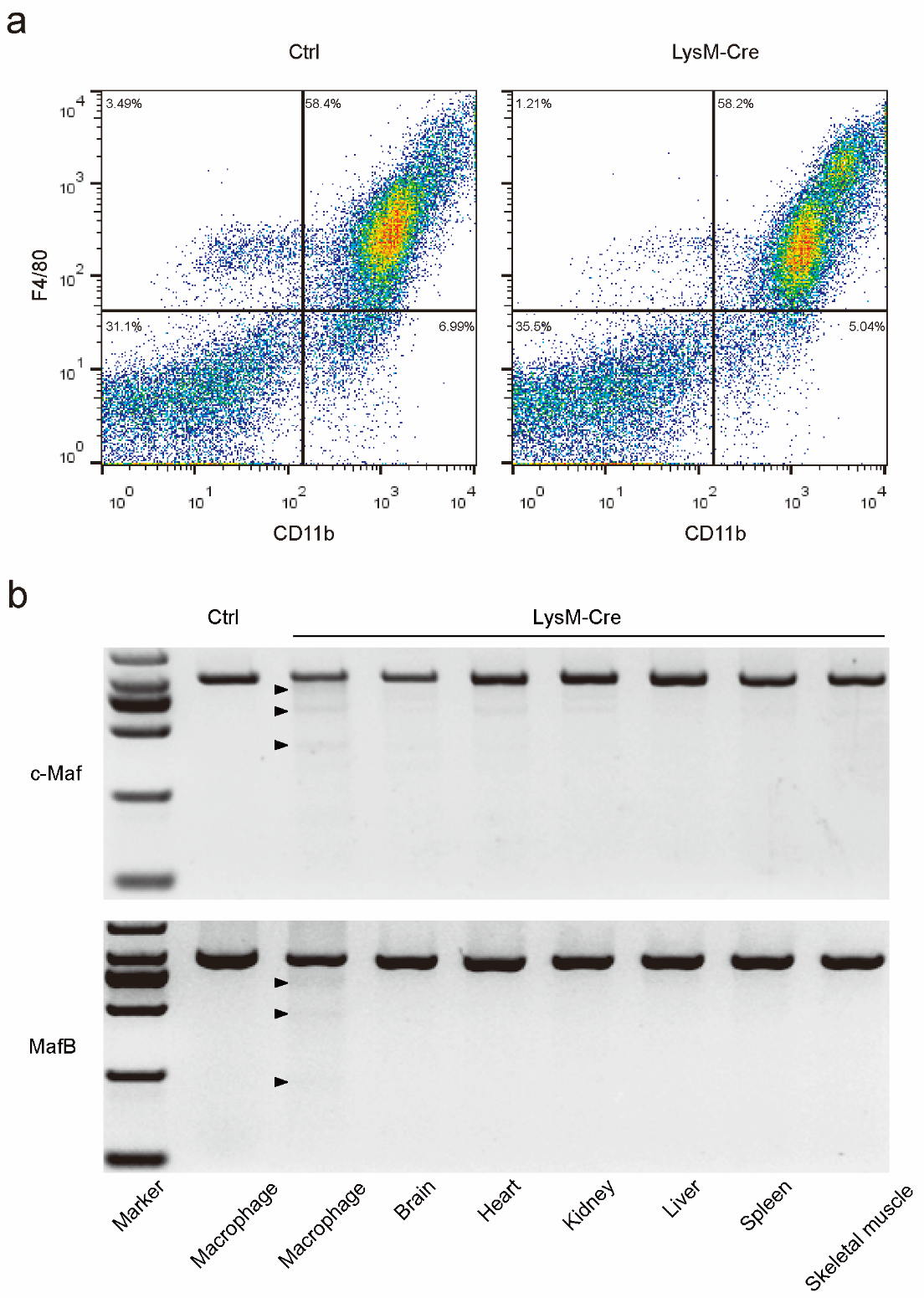
**

**Supplementary Fig. S2.** **Flow-cytometry and T7EN1 analysis of *c-Maf* and *MafB* mutations in macrophages of *sgRNAs^c-Maf/MafB^-LSL-Cas9;LysM-Cre* mice.** (a) Flow-cytometry of *c-Maf/MafB* double cKO transgenic mouse. Ctrl, *sgRNAs^c-Maf/MafB^-LSL-Cas9*; LysM-Cre, *sgRNAs^c-Maf/MafB^-LSL-Cas9;LysM-Cre*. (b) T7EN1 analysis of *c-Maf* and *MafB* mutations in tissues of *sgRNAs^c-Maf/MafB^-LSL-Cas9;LysM-Cre* mouse. Arrowheads pointed to the digested bands in macrophage genome sequences of Cre mice.


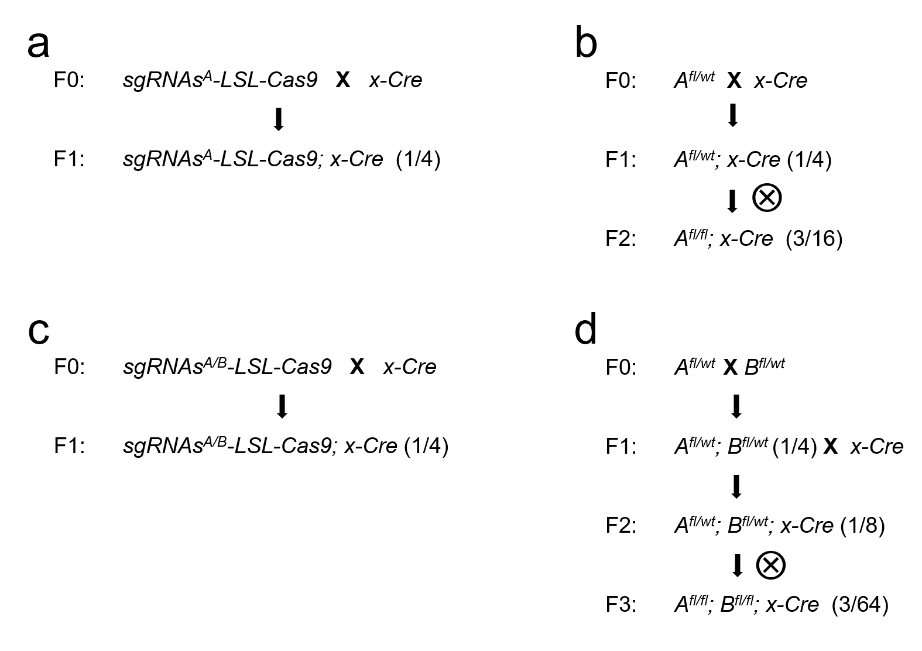


**Supplementary Fig. S3. Theoretical comparison of crossbreeding labor to generate cKO mice via sgRNAs-LSL-Cas9 and Cre-loxP strategies.** (a) Single gene cKO (x-dependent A gene deletion) can be achieved at generation F1 with 1/4 possibility via sgRNAs-LSL-Cas9 strategy. (b) Single gene cKO mice can be obtained at generation F2 with Cre-loxP system. (c) Double cKO (A and B genes) mice can be obtained at F1 with 1/4 possibility via sgRNAs-LSL-Cas9 strategy. (d) Using Cre-loxP strategy, double cKO can be achieved at F3 generation with lower possibility. ⮾, backcross.

| sgRNA | Sequence |
| --- | --- |
| EGFP sgRNA1 | GGTGGTGCAGATGAACTTCA |
| EGFP sgRNA2 | GCTTCATGTGGTCGGGGTAG |
| EGFP sgRNA3 | GGAGCGCACCATCTTCTTCA |
| EGFP sgRNA4 | GGCATCGACTTCAAGGAGGA |
|  |  |
| *c-Maf* sgRNA1 | GCACTGGCTGATGATGCGGT |
| *c-Maf* sgRNA2 | GTGGCTTCGATGGCTATGCG |
| *c-Maf* sgRNA3 | GCACTTCGACGACCGCTTCT |
|  |  |
| *MafB* sgRNA1 | GGTTCAGTCGGACTGAAGCT |
| *MafB* sgRNA2 | GGTGTGACTCACGATGACCT |
| *MafB* sgRNA3 | GTTCAGCTCACGCACCGACA |

**Supplementary Table S1. Sequences of sgRNAs targeting EGFP, *c-Maf* and *MafB*.**

| Sample | sgRNA1 | sgRNA2 | sgRNA3 | sgRNA4 |
| --- | --- | --- | --- | --- |
| 1 | 261bp deleted | | | |
| 2 | 261bp deleted | | | |
| 3 | 261bp deleted | | | |
| 4 | 261bp deleted | | | |
| 5 | 261bp deleted | | | |
| 6 | 262bp mutated | | | |
| 7 | 261bp deleted | | | |
| 8 | 261bp deleted | | | |
| 9 | 187bp mutated, 77bp deleted | | | 21bp deleted |
| 10 | 1bp deleted | 187bp deleted | | |
| 11 | 1bp mutated, 9bp deleted | × | × | × |
| 12 | × | × | × | 3bp deleted |
| 13 | × | × | × | 9bp deleted |
| 14 | × | × | × | 18bp deleted |
| 15 | × | × | × | 1bp deleted |
| 16 | × | × | × | 4bp deleted |
| 17 | × | × | × | 3bp deleted |
| 18 | × | × | × | × |
| 19 | × | × | × | × |
| 20 | × | × | × | × |
| 21 | × | × | × | × |
| 22 | × | × | × | × |
| 23 | × | × | × | × |
| Mutation Rate | | 73.91% | | |

“×”: No mutation in the relevant sgRNA targeting sites.

**Supplementary Table S2. Summary of EGFP sequences from the liver of *sgRNAs^EGFP^-LSL-Cas9;EGFP;Alb-Cre* mice.**

| Sample | sgRNA1 | sgRNA2 | sgRNA3 |
| --- | --- | --- | --- |
| 1 | 828bp deleted | | |
| 2 | 828bp deleted | | |
| 3 | 639bp deleted | | |
| 4 | 537bp deleted | | |
| 5 | 431bp mutated | | |
| 6 | 1bp deleted | 3bp deleted | 1bp deleted |
| 7 | 1bp deleted | 3bp deleted | 1bp deleted |
| 8 | 1bp deleted | 5bp deleted | 8bp deleted |
| 9 | 1bp deleted | 17bp deleted | 6bp deleted |
| 10 | 35bp deleted | 128bp deleted | 12bp deleted |
| 11 | 6bp deleted | 2bp deleted | 8bp deleted |
| 12 | 1bp deleted | 1bp deleted | 4bp deleted |
| 13 | × | 29bp deleted | 16bp deleted |
| 14 | × | 2bp deleted | 4bp deleted |
| 15 | 1bp mutated | × | × |
| 16 | × | 3bp mutated | × |
| 17 | × | 1bp mutated | × |
| 18 | × | 2bp inserted | × |
| 19 | × | × | 4bp deleted |
| 20 | × | × | × |
| 21 | × | × | × |
| 22 | × | × | × |
| 23 | × | × | × |
| 24 | × | × | × |
| 25 | × | × | × |
| 26 | × | × | × |
| 27 | × | × | × |
| 28 | × | × | × |
| 29 | × | × | × |
| 30 | × | × | × |
| 31 | × | × | × |
| 32 | × | × | × |
| 33 | × | × | × |
| Mutated rate | | 57.58% | |

“×”: No mutation in the relevant sgRNA targeting sites.

**Supplementary Table S3. Summary of *c-Maf* sequences from macrophages of *sgRNAs^c-Maf/MafB^-LSL-Cas9;LysM-Cre* mice.**

| Sample | sgRNA1 | sgRNA2 | sgRNA3 |
| --- | --- | --- | --- |
| 1 | 132bp deleted | 7bp deleted | 119 deleted |
| 2 | 2bp deleted | 1bp inserted | 227bp deleted |
| 3 | 1bp deleted | 5bp deleted | 424bp deleted |
| 4 | 2bp deleted | 2bp deleted | 1bp mutated; 42bp deleted |
| 5 | 130bp deleted | 159bp deleted | 301bp deleted |
| 6 | 9bp deleted | 2bp deleted | 57bp deleted |
| 7 | 9bp deleted | 47bp deleted | 84bp deleted |
| 8 | 110bp deleted | 95bp deleted | 69bp deleted |
| 9 | 123bp deleted | 250bp deleted | 178bp deleted |
| 10 | 2bp deleted | 5bp deleted | × |
| 11 | 1bp deleted | 5bp deleted | × |
| 12 | × | 1bp inserted | 21bp deleted |
| 13 | × | 4bp deleted | 5bp deleted |
| 14 | × | 5bp deleted | 4bp deleted |
| 15 | × | 5bp deleted | 4bp deleted |
| 16 | 1bp inserted | × | × |
| 17 | 1bp mutated | × | × |
| 18 | 83bp deleted | × | × |
| 19 | × | 12bp deleted | × |
| Mutated rate | | 100.00% | |

“×”: No mutation in the relevant sgRNA targeting sites.

**Supplementary Table S4. Summary of *MafB* sequences from macrophages of *sgRNAs^c-Maf/MafB^-LSL-Cas9;LysM-Cre* mice.**

| Primer | Sequence |
| --- | --- |
| EGFP-arr-for1 | ATGCGTCTCAACCGGTGGTGCAGATGAACTTCAGTTTTAGAGCTAGAAATAGCAAG |
| EGFP-arr-rev1 | ATGCGTCTCGTAAAACCTACCCCGACCACATGAAGCGGTGTTTCGTCCTTTCCACAAG |
|  |  |
| EGFP-arr-for2 | ATGCGTCTCATTTAGAGCTAGAAATAGCAAGTTAAAATAAG |
| EGFP-arr-rev2 | ATGCGTCTCGGCTCTAAAACTGAAGAAGATGGTGCGCTCCGGTGTTTCGTCCTTTCCACAAG |
|  |  |
| EGFP-arr-for3 | ATGCGTCTCAGAGCTAGAAATAGCAAGTTAAAATAAGGC |
| EGFP-arr-rev3 | ATGCGTCTCGAAACTCCTCCTTGAAGTCGATGCCGGTGTTTCGTCCTTTCCACAAG |
|  |  |
| Maf-arr-for1 | ATGCGTCTCAACCGCACTGGCTGATGATGCGGTGTTTTAGAGCTAGAAATAGCAAG |
| Maf-arr-rev1 | ATGCGTCTCGTAAAACAGCTTCAGTCCGACTGAACCGGTGTTTCGTCCTTTCCACAAG |
|  |  |
| Maf-arr-for2 | ATGCGTCTCATTTAGAGCTAGAAATAGCAAGTTAAAATAAG |
| Maf-arr-rev2 | ATGCGTCTCGGCTCTAAAACCGCATAGCCATCGAAGCCACGGTGTTTCGTCCTTTCCACAAG |
|  |  |
| Maf-arr-for3 | ATGCGTCTCAGAGCTAGAAATAGCAAGTTAAAATAAGGC |
| Maf-arr-rev3 | ATGCGTCTCGTCTAGCTCTAAAACAGGTCATCGTGAGTCACACCGGTGTTTCGTCCTTTCCACAAG |
|  |  |
| Maf-arr-for4 | ATGCGTCTCATAGAAATAGCAAGTTAAAATAAGGCTAG |
| Maf-arr-rev4 | ATGCGTCTCGCTATTTCTAGCTCTAAAACAGAAGCGGTCGTCGAAGTGCGGTGTTTCGTCCTTTCCACAAG |
|  |  |
| Maf-arr-for5 | ATGCGTCTCAATAGCAAGTTAAAATAAGGCTAGTCC |
| Maf-arr-rev5 | ATGCGTCTCGAAACTGTCGGTGCGTGAGCTGAACGGTGTTTCGTCCTTTCCACAAG |

**Supplementary Table S5. Primers for PCR amplification of the coding regions of U6-sgRNAs for EGFP, *c-Maf* and *MafB* from pUC57-sgRNA template.** Primers EGFP-arr-for1 and rev1, EGFP-arr-for2 and rev2, EGFP-arr-for3 and -rev3 were used to amplify segments contain EGFP sgRNA1 and2, EGFP sgRNA3, EGFP sgRNA4, respectively. Primers Maf-arr-for1 and rev1, Maf-arr-for2 and rev2, Maf-arr-for3 and rev3, Maf-arr-for4 and rev4, Maf-arr-for5 and rev5 were used to amplify segments contain *c-Maf* sgRNA1 and *MafB* sgRNA1, *c-Maf* sgRNA2, *MafB* sgRNA2, *c-Maf* sgRNA3, *MafB* sgRNA3, respectively.

pUC57-sgRNA:

TCGCGCGTTTCGGTGATGACGGTGAAAACCTCTGACACATGCAGCTCCCGGAGACGGTCACAGCTTGTCTGTAAGCGGATGCCGGGAGCAGACAAGCCCGTCAGGGCGCGTCAGCGGGTGTTGGCGGGTGTCGGGGCTGGCTTAACTATGCGGCATCAGAGCAGATTGTACTGAGAGTGCACCATATGCGGTGTGAAATACCGCACAGATGCGTAAGGAGAAAATACCGCATCAGGCGCCATTCGCCATTCAGGCTGCGCAACTGTTGGGAAGGGCGATCGGTGCGGGCCTCTTCGCTATTACGCCAGCTGGCGAAAGGGGGATGTGCTGCAAGGCGATTAAGTTGGGTAACGCCAGGGTTTTCCCAGTCACGACGTTGTAAAACGACGGCCAGTGAATTCGAGCTCGGTACCTCGCGAATGCATCTAGATATCGGATCCCTAATACGACTCACTATAGGTGAGACCGAGAGAGGGTCTCAGTTTTAGAGCTAGAAATAGCAAGTTAAAATAAGGCTAGTCCGTTATCAACTTGAAAAAGTGGCACCGAGTCGGTGCTTTTTTTAAAGGGCCCGTCGAATGCAGAGGCCTGCATGCAAGCTTGGCGTAATCATGTTCCTGTGTGAAATTGTTATCCGCTCACAATTCCACACAACTAGCTCACCGAGGGCCTATTTCCCATGATTCCTTCATATTTGCATATACGATACAAGGCTGTTAGAGAGATAATTGGAATTAATTTGACTGTAAACACAAAGATATTAGTACAAAATACGTGACGTAGAAAGTAATAATTTCTTGGGTAGTTTGCAGTTTTAAAATTATGTTTTAAAATGGACTATCATATGCTTACCGTAACTTGAAAGTATTTCGATTTCTTGGCTTTATATATCTTGTGGAAAGGACGAAACACCGCATAAAGTGTAAAGCCTGGGGTGCCTAATGAGTGAGCTAACTCACATTAATTGCGTTGCGCTCACTGCCCGCTTTCCAGTCGGGAAACCTGTCGTGCCAGCTGCATTAATGAATCGGCCAACGCGCGGGGAGAGGCGGTTTGCGTATTGGGCGCGGCCGCCGCTTCCTCGCTCACTGACTCGCTGCGCTCGGTCGTTCGGCTGCGGCGAGCGGTATCAGCTCACTCAAAGGCGGTAATACGGTTATCCACAGAATCAGGGGATAACGCAGGAAAGAACATGTGAGCAAAAGGCCAGCAAAAGGCCAGGAACCGTAAAAAGGCCGCGTTGCTGGCGTTTTTCCATAGGCTCCGCCCCCCTGACGAGCATCACAAAAATCGACGCTCAAGTCAGAGGTGGCGAAACCCGACAGGACTATAAAGATACCAGGCGTTTCCCCCTGGAAGCTCCCTCGTGCGCTCTCCTGTTCCGACCCTGCCGCTTACCGGATACCTGTCCGCCTTTCTCCCTTCGGGAAGCGTGGCGCTTTCTCATAGCTCACGCTGTAGGTATCTCAGTTCGGTGTAGGTCGTTCGCTCCAAGCTGGGCTGTGTGCACGAACCCCCCGTTCAGCCCGACCGCTGCGCCTTATCCGGTAACTATCGTCTTGAGTCCAACCCGGTAAGACACGACTTATCGCCACTGGCAGCAGCCACTGGTAACAGGATTAGCAGAGCGAGGTATGTAGGCGGTGCTACAGAGTTCTTGAAGTGGTGGCCTAACTACGGCTACACTAGAAGAACAGTATTTGGTATCTGCGCTCTGCTGAAGCCAGTTACCTTCGGAAAAAGAGTTGGTAGCTCTTGATCCGGCAAACAAACCACCGCTGGTAGCGGTGGTTTTTTTGTTTGCAAGCAGCAGATTACGCGCAGAAAAAAAGGATCTCAAGAAGATCCTTTGATCTTTTCTACGGGGTCTGACGCTCAGTGGAACGAAAACTCACGTTAAGGGATTTTGGTCATGAGATTATCAAAAAGGATCTTCACCTAGATCCTTTTAAATTAAAAATGAAGTTTTAAATCAATCTAAAGTATATATGAGTAAACTTGGTCTGACAGTTAGAAAAACTCATCGAGCATCAAATGAAACTGCAATTTATTCATATCAGGATTATCAATACCATATTTTTGAAAAAGCCGTTTCTGTAATGAAGGAGAAAACTCACCGAGGCAGTTCCATAGGATGGCAAGATCCTGGTATCGGTCTGCGATTCCGACTCGTCCAACATCAATACAACCTATTAATTTCCCCTCGTCAAAAATAAGGTTATCAAGTGAGAAATCACCATGAGTGACGACTGAATCCGGTGAGAATGGCAAAAGTTTATGCATTTCTTTCCAGACTTGTTCAACAGGCCAGCCATTACGCTCGTCATCAAAATCACTCGCATCAACCAAACCGTTATTCATTCGTGATTGCGCCTGAGCGAGACGAAATACGCGATCGCTGTTAAAAGGACAATTACAAACAGGAATCGAATGCAACCGGCGCAGGAACACTGCCAGCGCATCAACAATATTTTCACCTGAATCAGGATATTCTTCTAATACCTGGAATGCTGTTTTCCCAGGGATCGCAGTGGTGAGTAACCATGCATCATCAGGAGTACGGATAAAATGCTTGATGGTCGGAAGAGGCATAAATTCCGTCAGCCAGTTTAGTCTGACCATCTCATCTGTAACATCATTGGCAACGCTACCTTTGCCATGTTTCAGAAACAACTCTGGCGCATCGGGCTTCCCATACAATCGATAGATTGTCGCACCTGATTGCCCGACATTATCGCGAGCCCATTTATACCCATATAAATCAGCATCCATGTTGGAATTTAATCGCGGCCTAGAGCAAGACGTTTCCCGTTGAATATGGCTCATACTCTTCCTTTTTCAATATTATTGAAGCATTTATCAGGGTTATTGTCTCATGAGCGGATACATATTTGAATGTATTTAGAAAAATAAACAAATAGGGGTTCCGCGCACATTTCCCCGAAAAGTGCCACCTGACGTCTAAGAAACCATTATTATCATGACATTAACCTATAAAAATAGGCGTATCACGAGGCCCTTTCGTC

pU6-*BsmBI*-UbC-Cas9:

TCTAGAGTCGATGACGCGTGAGGGCCTATTTCCCATGATTCCTTCATATTTGCATATACGATACAAGGCTGTTAGAGAGATAATTGGAATTAATTTGACTGTAAACACAAAGATATTAGTACAAAATACGTGACGTAGAAAGTAATAATTTCTTGGGTAGTTTGCAGTTTTAAAATTATGTTTTAAAATGGACTATCATATGCTTACCGTAACTTGAAAGTATTTCGATTTCTTGGCTTTATATATCTTGTGGAAAGGACGAAACACCGGGAGACGGAGAGAGCGTCTCCGTTTTAGAGCTAGAAATAGCAAGTTAAAATAAGGCTAGTCCGTTATCAACTTGAAAAAGTGGCACCGAGTCGGTGCTTTTTTTAAAGGGCCCACATAGCTTCGACTGCAGAGGCCTGCATGCAAGCTTGGCGTAATCATAAATTCTCGACCTCGAGACAAATGGCAGTATTCATCCACAACGCGTGGCCTCCGCGCCGGGTTTTGGCGCCTCCCGCGGGCGCCCCCCTCCTCACGGCGAGCGCTGCCACGTCAGACGAAGGGCGCAGCGAGCGTCCTGATCCTTCCGCCCGGACGCTCAGGACAGCGGCCCGCTGCTCATAAGACTCGGCCTTAGAACCCCAGTATCAGCAGAAGGACATTTTAGGACGGGACTTGGGTGACTCTAGGGCACTGGTTTTCTTTCCAGAGAGCGGAACAGGCGAGGAAAAGTAGTCCCTTCTCGGCGATTCTGCGGAGGGATCTCCGTGGGGCGGTGAACGCCGATGATTATATAAGGACGCGCCGGGTGTGGCACAGCTAGTTCCGTCGCAGCCGGGATTTGGGTCGCGGTTCTTGTTTGTGGATCGCTGTGATCGTCACTTGGTGAGTAGCGGGCTGCTGGGCTGGCCGGGGCTTTCGTGGCCGCCGGGCCGCTCGGTGGGACGGAAGCGTGTGGAGAGACCGCCAAGGGCTGTAGTCTGGGTCCGCGAGCAAGGTTGCCCTGAACTGGGGGTTGGGGGGAGCGCAGCAAAATGGCGGCTGTTCCCGAGTCTTGAATAGAACCTTCGCTAATGCGGGAAAGCTCTTATTCGGGTGAGATGGGCTGGGGCACCATCTGGGGACCCTGACGTGAAGTTTGTCACTGACTGGAGAACTCGGTTTGTCGTCTGTTGCGGGGGCGGCAGTTATGGCGGTGCCGTTGGGCAGTGCACCCGTACCTTTGGGAGCGCGCGCCCTCGTCGTGTCGTGACGTCACCCGTTCTGTTGGCTTATAATGCAGGGTGGGGCCACCTGCCGGTAGGTGTGCGGTAGGCTTTTCTCCGTCGCAGGACGCAGGGTTCGGGCCTAGGGTAGGCTCTCCTGAATCGACAGGCGCCGGACCTCTGGTGAGGGGAGGGATAAGTGAGGCGTCAGTTTCTTTGGTCGGTTTTATGTACCTATCTTCTTAAGTAGCTGAAGCTCCGGTTTTGAACTATGCGCTCGGGGTTGGCGAGTGTGTTTTGTGAAGTTTTTTAGGCACCTTTTGAAATGTAATCATTTGGGTCAATATGTAATTTTCAGTGTTAGACTAGTAAATTGTCCGCTAAATTCTGGCCGTTTTTGGCTTTTTTGTTAGACCTAGTGATAACTTCGTATAGCATACATTATACGAAGTTATCTAGTCGCCACCATGGCCTCCTCCGAGGACGTCATCAAGGAGTTCATGCGCTTCAAGGTGCGCATGGAGGGCTCCGTGAACGGCCACGAGTTCGAGATCGAGGGCGAGGGCGAGGGCCGCCCCTACGAGGGCACCCAGACCGCCAAGCTGAAGGTGACCAAGGGCGGCCCCCTGCCCTTCGCCTGGGACATCCTGTCCCCCCAGTTCCAGTACGGCTCCAAGGTGTACGTGAAGCACCCCGCCGACATCCCCGACTACAAGAAGCTGTCCTTCCCCGAGGGCTTCAAGTGGGAGCGCGTGATGAACTTCGAGGACGGCGGCGTGGTGACCGTGACCCAGGACTCCTCCCTGCAGGACGGCTGCTTCATCTACAAGGTGAAGTTCATCGGCGTGAACTTCCCCTCCGACGGCCCCGTAATGCAGAAGAAGACTATGGGCTGGGAGCCCTCCACCGAGCGCCTGTACCCCCGCGACGGCGTGCTGAAGGGCGAGATCCACAAGGCCCTGAAGCTGAAGGACGGCGGCCACTACCTGGTGGAGTTCAAGTCCATCTACATGGCCAAGAAGCCCGTGCAGCTGCCCGGCTACTACTACGTGGACTCCAAGCTGGACATCACCTCCCACAACGAGGACTACACCATCGTGGAGCAGTACGAGCGCACCGAGGGCCGCCACCACCTGTTCCTGTAGCGGCCCGGGCTGCAGGAATTCTGTGCCTTCTAGTTGCCAGCCATCTGTGTTTGCCCCTCCCCCGTGCCTTCCTTGACCCTGGAACGTGCCACTCCCCACTGTCCTTTCCCTAATAAAATGAGGAAATTGCATCGCATTGTCTGAGTAGGTGTCATTCTATTCTGGGGGGTGGGGTGGGGCAGGACAGCAAGGGGGAGGATTGGGAAGACAATAGCAGGCATGCTGGGGATGCGGTGGGCTCTATGGAATTCTGTGCCTTCTAGTTGCCAGCCATCTGTTGTTTGCCCCTCCCCCGTGCCTTCCTTGACCCTGGAAGGTGCCACTCCCACTGTCCTTTCCTAATAAAATGAGGAAATTGCATCGCATTGTCTGAGTAGGTGTCATTCTATTCTGGGGGGTGGGGTGGGGCAGGACAGCAAGGGGGAGGATTGGGAAGACAATAGCAGGCATGCTGGGGATGCGGTGGGCTCTATGGAATTCGATATCAAGCTTATCGATAACTTCGTATAGCATACATTATACGAAGTTATCGGATCACCGGTCACCATGGGACCTAAGAAAAAGAGGAAGGTGGCGGCCGCTGACTACAAGGATGACGACGATAAATCTAGAGACAAGAAATACTCTATTGGACTGGATATCGGGACAAACTCCGTTGGCTGGGCCGTCATAACCGACGAGTATAAGGTGCCAAGCAAGAAATTCAAGGTGCTGGGTAATACTGACCGCCATTCAATCAAGAAGAACCTGATCGGAGCACTCCTCTTCGACTCCGGTGAAACCGCTGAAGCTACTCGGCTGAAGCGGACCGCAAGGCGGAGATACACCCGCCGCAAGAATCGGATATGTTATCTGCAAGAGATCTTTAGCAACGAAATGGCTAAGGTGGACGACTCCTTCTTTCACCGCCTGGAAGAGAGCTTTCTGGTGGAGGAGGATAAGAAACACGAGAGGCACCCTATATTCGGAAATATCGTGGATGAGGTGGCTTACCATGAAAAGTATCCTACAATCTACCATCTGAGGAAGAAGCTGGTGGACAGCACCGATAAAGCAGACCTGAGGCTCATCTATCTGGCCCTGGCTCATATGATAAAGTTTAGAGGACACTTTCTGATCGAGGGCGACCTGAATCCCGATAATTCCGATGTGGATAAACTCTTCATTCAACTGGTGCAGACATATAACCAACTGTTCGAGGAGAATCCCATAAACGCTTCTGGTGTGGATGCCAAGGCTATTCTGTCCGCTCGGCTGTCCAAGTCACGCAGACTGGAGAATCTGATTGCCCAACTGCCAGGAGAAAAGAAGAACGGCCTGTTTGGGAACCTCATCGCCCTGAGCCTGGGCCTGACACCTAACTTCAAGTCCAATTTTGATCTGGCCGAAGATGCTAAACTCCAGCTCTCCAAGGACACCTATGACGATGATCTGGACAACCTGCTCGCACAGATAGGCGACCAGTACGCCGATCTCTTTCTGGCTGCTAAGAATCTCTCCGACGCCATTCTGCTGAGCGACATACTCCGGGTCAACACTGAGATCACCAAAGCACCTCTGAGCGCCTCCATGATAAAACGCTATGATGAACACCATCAAGACCTGACTCTGCTCAAAGCCCTCGTGAGGCAACAGCTGCCAGAGAAGTACAAAGAGATATTCTTCGACCAGAGCAAGAATGGATATGCCGGATACATCGATGGCGGAGCATCACAGGAAGAATTTTACAAGTTCATCAAACCAATCCTCGAGAAGATGGACGGTACTGAAGAGCTGCTGGTGAAGCTGAACAGGGAGGACCTGCTGAGGAAGCAGAGGACCTTTGATAATGGCTCCATTCCACATCAGATACACCTGGGAGAGCTGCATGCAATCCTCCGCAGGCAGGAGGATTTCTATCCTTTCCTGAAGGATAACCGGGAGAAGATAGAGAAGATCCTGACCTTCAGGATCCCTTATTACGTCGGCCCTCTGGCTAGAGGCAACTCCCGCTTCGCTTGGATGACCAGGAAATCTGAGGAGACAATTACTCCTTGGAACTTCGAAGAGGTCGTGGATAAGGGCGCAAGCGCCCAGTCATTCATCGAACGGATGACCAATTTCGATAAGAACCTGCCCAACGAGAAGGTCCTGCCCAAACATTCACTCCTGTACGAGTATTTCACCGTCTATAACGAGCTGACTAAAGTGAAGTACGTGACCGAGGGCATGAGGAAGCCTGCCTTCCTGTCCGGAGAGCAGAAGAAGGCTATCGTTGATCTGCTCTTCAAGACTAATAGAAAGGTGACAGTGAAGCAGCTCAAGGAGGATTACTTTAAGAAGATCGAATGCTTTGACTCAGTGGAAATCTCTGGCGTGGAGGACCGCTTTAATGCCAGCCTGGGCACTTACCATGATCTGCTGAAGATAATCAAAGACAAAGATTTCCTCGATAATGAGGAGAACGAGGACATCCTGGAAGATATCGTGCTGACCCTGACTCTGTTCGAGGATAGAGAGATGATCGAAGAGCGCCTGAAGACCTATGCCCATCTGTTTGACGATAAAGTCATGAAACAGCTCAAGCGGCGGCGCTACACTGGGTGGGGTAGACTCTCCAGGAAACTCATAAACGGCATCCGCGACAAACAGAGCGGAAAGACCATCCTGGATTTCCTGAAATCCGACGGATTCGCTAACAGGAACTTCATGCAACTGATTCACGATGACTCTCTGACATTTAAAGAGGACATCCAGAAGGCACAGGTGAGCGGTCAAGGCGACAGCCTGCACGAGCACATCGCCAACCTCGCTGGATCACCCGCCATAAAGAAGGGAATACTGCAGACAGTCAAGGTCGTGGACGAACTCGTCAAAGTGATGGGTCGGCACAAGCCAGAGAATATCGTTATCGAAATGGCAAGGGAGAACCAAACCACCCAGAAGGGCCAGAAGAACTCTCGGGAACGGATGAAAAGAATCGAAGAGGGAATTAAGGAGCTGGGATCTCAGATACTGAAGGAGCACCCTGTGGAGAATACACAGCTCCAGAACGAGAAACTCTACCTGTACTACCTCCAGAACGGGCGGGACATGTACGTTGACCAGGAACTCGACATCAACCGGCTGTCCGATTATGACGTGGACCATATTGTTCCACAGTCCTTCCTCAAAGATGACTCCATTGACAACAAGGTGCTGACCAGATCCGATAAGAATCGCGGTAAGTCTGACAATGTTCCATCAGAAGAGGTGGTCAAGAAGATGAAGAATTACTGGCGGCAGCTCCTCAACGCCAAACTGATCACCCAGCGGAAGTTTGACAATCTGACTAAGGCAGAAAGAGGAGGTCTGAGCGAACTCGACAAGGCCGGCTTTATTAAGAGGCAACTGGTCGAAACACGCCAGATTACCAAACACGTGGCACAAATCCTCGACTCTAGGATGAACACTAAGTACGATGAGAACGATAAGCTGATCAGGGAAGTGAAAGTGATAACTCTGAAGAGCAAGCTGGTGTCTGACTTCCGGAAGGACTTTCAATTCTACAAAGTTCGCGAAATAAACAATTACCATCATGCTCACGATGCCTATCTCAATGCTGTCGTTGGCACCGCCCTGATCAAGAAATACCCTAAACTGGAGTCTGAGTTCGTGTACGGTGACTATAAAGTCTACGATGTGAGGAAGATGATAGCAAAGTCTGAGCAAGAGATTGGCAAAGCCACCGCCAAGTACTTCTTCTACTCTAATATCATGAATTTCTTTAAGACTGAGATAACCCTGGCTAACGGCGAAATCCGGAAGCGCCCACTGATCGAAACAAACGGAGAAACAGGAGAAATCGTGTGGGATAAAGGCAGGGACTTCGCAACTGTGCGGAAGGTGCTGTCCATGCCACAAGTCAATATCGTGAAGAAGACCGAAGTGCAGACCGGCGGATTCTCAAAGGAGAGCATCCTGCCAAAGCGGAACTCTGACAAGCTGATCGCCAGGAAGAAAGATTGGGACCCAAAGAAGTATGGCGGTTTCGATTCCCCTACAGTGGCTTATTCCGTTCTGGTCGTGGCAAAAGTGGAGAAAGGCAAGTCCAAGAAACTCAAGTCTGTTAAGGAGCTGCTCGGAATTACTATTATGGAGAGATCCAGCTTCGAGAAGAATCCAATCGATTTCCTGGAAGCTAAGGGCTATAAAGAAGTGAAGAAAGATCTCATCATCAAACTGCCCAAGTACTCTCTCTTTGAGCTGGAGAATGGTAGGAAGCGGATGCTGGCCTCCGCCGGAGAGCTGCAGAAAGGAAACGAGCTGGCTCTGCCCTCCAAATACGTGAACTTCCTGTATCTGGCCTCCCACTACGAGAAACTCAAAGGTAGCCCTGAAGACAATGAGCAGAAGCAACTCTTTGTTGAGCAACATAAACACTACCTGGACGAAATCATTGAACAGATTAGCGAGTTCAGCAAGCGGGTTATTCTGGCCGATGCAAACCTCGATAAAGTGCTGAGCGCATATAATAAGCACAGGGACAAGCCAATTCGCGAACAAGCAGAGAATATTATCCACCTCTTTACTCTGACTAATCTGGGCGCTCCTGCTGCCTTCAAGTATTTCGATACAACTATTGACAGGAAGCGGTACACCTCTACCAAAGAAGTTCTCGATGCCACCCTGATACACCAGTCAATTACCGGACTGTACGAGACTCGCATCGACCTGTCTCAGCTCGGCGGCGACGGTTCTCCCAAGAAGAAGAGGAAAGTCTCGAGCGGTGGAGCTGCAGGATAGGGTACCAGCTCGCTGATCAGCCTCGACTGTGCCTTCTAGTTGCCAGCCATCTGTTGTTTGCCCCTCCCCCGTGCCTTCCTTGACCCTGGAAGGTGCCACTCCCACTGTCCTTTCCTAATAAAATGAGGAAATTGCATCGCATTGTCTGAGTAGGTGTCATTCTATTCTGGGGGGTGGGGTGGGGCAGGACAGCAAGGGGGAGGATTGGGAAGACAATAGCAGGCATGCTGGGGATGCGGTGGGCTCTATGGCTTCTGAGGCGGAAAGAACCAGCTGGGGCTCGACTCTAGAGGGACAGCCCCCCCCCAAAGCCCCCAGGGATGTAATTACGTCCCTCCCCCGCTAGGGGGCAGCAGCGAGCCGCCCGGGGCTCCGCTCCGGTCCGGCGCTCCCCCCGCATCCCCGAGCCGGCAGCGTGCGGGGACAGCCCGGGCACGGGGAAGGTGGCACGGGATCGCTTTCCTCTGAACGCTTCTCGCTGCTCTTTGAGCCTGCAGACACCTGGGGGGATACGGGGAAAAAGCTTTAGGCTGAAAGAGAGATTTAGAATGACAGAATCATAGAACGGCCTGGGTTGCAAAGGAGCACAGTGCTCATCCAGATCCAACCCCCTGCTATGTGCAGGGTCATCAACCAGCAGCCCAGGCTGCCCAGAGCCACATCCAGCCTGGCCTTGAATGCCTGCAGGGATGGGGCATCCACAGCCTCCTTGGGCAACCTGTTCAGTGCGTCACCACCCTCTGGGGGAAAAACTGCCTCCTCATATCCAACCCAAACCTCCCCTGTCTCAGTGTAAAGCCATTCCCCCTTGTCCTATCAAGGGGGAGTTTGCTGTGACATTGTTGGTCTGGGGTGACACATGTTTGCCAATTCAGTGCATCACGGAGAGGCAGATCTTGGGGATAAGGAAGTGCAGGACAGCATGGACGTGGGACATGCAGGTGTTGAGGGCTCTGGGACACTCTCCAAGTCACAGCGTTCAGAACAGCCTTAAGGATAAGAAGATAGGATAGAAGGACAAAGAGCAAGTTAAAACCCAGCATGGAGAGGAGCACAAAAAGGCCACAGACACTGCTGGTCCCTGTGTCTGAGCCTGCATGTTTGATGGTGTCTGGATGCAAGCAGAAGGGGTGGAAGAGCTTGCCTGGAGAGATACAGCTGGGTCAGTAGGACTGGGACAGGCAGCTGGAGAATTGCCATGTAGATGTTCATACAATCGTCAAATCATGAAGGCTGGAAAAGCCCTCCAAGATCCCCAAGACCAACCCCAACCCACCCACCGTGCCCACTGGCCATGTCCCTCAGTGCCACATCCCCACAGTTCTTCATCACCTCCAGGGACGGTGACCCCCCCACCTCCGTGGGCAGCTGTGCCACTGCAGCACCGCTCTTTGGAGAAGGTAAATCTTGCTAAATCCAGCCCGACCCTCCCCTGGCACAACGTAAGGCCATTATCTCTCATCCAACTCCAGGACGGAGTCAGTGAGGATGGGGCTCTAGAGGGACAGCCCCCCCCCAAAGCCCCCAGGGATGTAATTACGTCCCTCCCCCGCTAGGGGGCAGCAGCGAGCCGCCCGGGGCTCCGCTCCGGTCCGGCGCTCCCCCCGCATCCCCGAGCCGGCAGCGTGCGGGGACAGCCCGGGCACGGGGAAGGTGGCACGGGATCGCTTTCCTCTGAACGCTTCTCGCTGCTCTTTGAGCCTGCAGACACCTGGGGGGATACGGGGAAAAAGCTTTAGGCTGAAAGAGAGATTTAGAATGACAGAATCATAGAACGGCCTGGGTTGCAAAGGAGCACAGTGCTCATCCAGATCCAACCCCCTGCTATGTGCAGGGTCATCAACCAGCAGCCCAGGCTGCCCAGAGCCACATCCAGCCTGGCCTTGAATGCCTGCAGGGATGGGGCATCCACAGCCTCCTTGGGCAACCTGTTCAGTGCGTCACCACCCTCTGGGGGAAAAACTGCCTCCTCATATCCAACCCAAACCTCCCCTGTCTCAGTGTAAAGCCATTCCCCCTTGTCCTATCAAGGGGGAGTTTGCTGTGACATTGTTGGTCTGGGGTGACACATGTTTGCCAATTCAGTGCATCACGGAGAGGCAGATCTTGGGGATAAGGAAGTGCAGGACAGCATGGACGTGGGACATGCAGGTGTTGAGGGCTCTGGGACACTCTCCAAGTCACAGCGTTCAGAACAGCCTTAAGGATAAGAAGATAGGATAGAAGGACAAAGAGCAAGTTAAAACCCAGCATGGAGAGGAGCACAAAAAGGCCACAGACACTGCTGGTCCCTGTGTCTGAGCCTGCATGTTTGATGGTGTCTGGATGCAAGCAGAAGGGGTGGAAGAGCTTGCCTGGAGAGATACAGCTGGGTCAGTAGGACTGGGACAGGCAGCTGGAGAATTGCCATGTAGATGTTCATACAATCGTCAAATCATGAAGGCTGGAAAAGCCCTCCAAGATCCCCAAGACCAACCCCAACCCACCCACCGTGCCCACTGGCCATGTCCCTCAGTGCCACATCCCCACAGTTCTTCATCACCTCCAGGGACGGTGACCCCCCCACCTCCGTGGGCAGCTGTGCCACTGCAGCACCGCTCTTTGGAGAAGGTAAATCTTGCTAAATCCAGCCCGACCCTCCCCTGGCACAACGTAAGGCCATTATCTCTCATCCAACTCCAGGACGGAGTCAGTGAGGATGGGGCTCTAGAGGATCAGTGCATGCTGGGGATGCGGTGGGCTCTATGGCTTCTGAGGCGGAAAGAACCAGCTGCATTAATGAATCGGCCAACGCGCGGGGAGAGGCGGTTTGCGTATTGGGCGCTCTTCCGCTTCCTCGCTCACTGACTCGCTGCGCTCGGTCGTTCGGCTGCGGCGAGCGGTATCAGCTCACTCAAAGGCGGTAATACGGTTATCCACAGAATCAGGGGATAACGCAGGAAAGAACATGTGAGCAAAAGGCCAGCAAAAGGCCAGGAACCGTAAAAAGGCCGCGTTGCTGGCGTTTTTCCATAGGCTCCGCCCCCCTGACGAGCATCACAAAAATCGACGCTCAAGTCAGAGGTGGCGAAACCCGACAGGACTATAAAGATACCAGGCGTTTCCCCCTGGAAGCTCCCTCGTGCGCTCTCCTGTTCCGACCCTGCCGCTTACCGGATACCTGTCCGCCTTTCTCCCTTCGGGAAGCGTGGCGCTTTCTCAATGCTCACGCTGTAGGTATCTCAGTTCGGTGTAGGTCGTTCGCTCCAAGCTGGGCTGTGTGCACGAACCCCCCGTTCAGCCCGACCGCTGCGCCTTATCCGGTAACTATCGTCTTGAGTCCAACCCGGTAAGACACGACTTATCGCCACTGGCAGCAGCCACTGGTAACAGGATTAGCAGAGCGAGGTATGTAGGCGGTGCTACAGAGTTCTTGAAGTGGTGGCCTAACTACGGCTACACTAGAAGGACAGTATTTGGTATCTGCGCTCTGCTGAAGCCAGTTACCTTCGGAAAAAGAGTTGGTAGCTCTTGATCCGGCAAACAAACCACCGCTGGTAGCGGTGGTTTTTTTGTTTGCAAGCAGCAGATTACGCGCAGAAAAAAAGGATCTCAAGAAGATCCTTTGATCTTTTCTACGGGGTCTGACGCTCAGTGGAACGAAAACTCACGTTAAGGGATTTTGGTCATGAGATTATCAAAAAGGATCTTCACCTAGATCCTTTTAAATTAAAAATGAAGTTTTAAATCAATCTAAAGTATATATGAGTAAACTTGGTCTGACAGTTACCAATGCTTAATCAGTGAGGCACCTATCTCAGCGATCTGTCTATTTCGTTCATCCATAGTTGCCTGACTCCCCGTCGTGTAGATAACTACGATACGGGAGGGCTTACCATCTGGCCCCAGTGCTGCAATGATACCGCGAGACCCACGCTCACCGGCTCCAGATTTATCAGCAATAAACCAGCCAGCCGGAAGGGCCGAGCGCAGAAGTGGTCCTGCAACTTTATCCGCCTCCATCCAGTCTATTAATTGTTGCCGGGAAGCTAGAGTAAGTAGTTCGCCAGTTAATAGTTTGCGCAACGTTGTTGCCATTGCTACAGGCATCGTGGTGTCACGCTCGTCGTTTGGTATGGCTTCATTCAGCTCCGGTTCCCAACGATCAAGGCGAGTTACATGATCCCCCATGTTGTGCAAAAAAGCGGTTAGCTCCTTCGGTCCTCCGATCGTTGTCAGAAGTAAGTTGGCCGCAGTGTTATCACTCATGGTTATGGCAGCACTGCATAATTCTCTTACTGTCATGCCATCCGTAAGATGCTTTTCTGTGACTGGTGAGTACTCAACCAAGTCATTCTGAGAATAGTGTATGCGGCGACCGAGTTGCTCTTGCCCGGCGTCAATACGGGATAATACCGCGCCACATAGCAGAACTTTAAAAGTGCTCATCATTGGAAAACGTTCTTCGGGGCGAAAACTCTCAAGGATCTTACCGCTGTTGAGATCCAGTTCGATGTAACCCACTCGTGCACCCAACTGATCTTCAGCATCTTTTACTTTCACCAGCGTTTCTGGGTGAGCAAAAACAGGAAGGCAAAATGCCGCAAAAAAGGGAATAAGGGCGACACGGAAATGTTGAATACTCATACTCTTCCTTTTTCAATATTATTGAAGCATTTATCAGGGTTATTGTCTCATGAGCGGATACATATTTGAATGTATTTAGAAAAATAAACAAATAGGGGTTCCGCGCACATTTCCCCGAAAAGTGCCACCTGACGTCGACGGATCGGGAGATCCTCTAGAGCCCCATCCTCACTGACTCCGTCCTGGAGTTGGATGAGAGATAATGGCCTTACGTTGTGCCAGGGGAGGGTCGGGCTGGATTTAGCAAGATTTACCTTCTCCAAAGAGCGGTGCTGCAGTGGCACAGCTGCCCACGGAGGTGGGGGGGTCACCGTCCCTGGAGGTGATGAAGAACTGTGGGGATGTGGCACTGAGGGACATGGCCAGTGGGCACGGTGGGTGGGTTGGGGTTGGTCTTGGGGATCTTGGAGGGCTTTTCCAGCCTTCATGATTTGACGATTGTATGAACATCTACATGGCAATTCTCCAGCTGCCTGTCCCAGTCCTACTGACCCAGCTGTATCTCTCCAGGCAAGCTCTTCCACCCCTTCTGCTTGCATCCAGACACCATCAAACATGCAGGCTCAGACACAGGGACCAGCAGTGTCTGTGGCCTTTTTGTGCTCCTCTCCATGCTGGGTTTTAACTTGCTCTTTGTCCTTCTATCCTATCTTCTTATCCTTAAGGCTGTTCTGAACGCTGTGACTTGGAGAGTGTCCCAGAGCCCTCAACACCTGCATGTCCCACGTCCATGCTGTCCTGCACTTCCTTATCCCCAAGATCTGCCTCTCCGTGATGCACTGAATTGGCAAACATGTGTCACCCCAGACCAACAATGTCACAGCAAACTCCCCCTTGATAGGACAAGGGGGAATGGCTTTACACTGAGACAGGGGAGGTTTGGGTTGGATATGAGGAGGCAGTTTTTCCCCCAGAGGGTGGTGACGCACTGAACAGGTTGCCCAAGGAGGCTGTGGATGCCCCATCCCTGCAGGCATTCAAGGCCAGGCTGGATGTGGCTCTGGGCAGCCTGGGCTGCTGGTTGATGACCCTGCACATAGCAGGGGGTTGGATCTGGATGAGCACTGTGCTCCTTTGCAACCCAGGCCGTTCTATGATTCTGTCATTCTAAATCTCTCTTTCAGCCTAAAGCTTTTTCCCCGTATCCCCCCAGGTGTCTGCAGGCTCAAAGAGCAGCGAGAAGCGTTCAGAGGAAAGCGATCCCGTGCCACCTTCCCCGTGCCCGGGCTGTCCCCGCACGCTGCCGGCTCGGGGATGCGGGGGGAGCGCCGGACCGGAGCGGAGCCCCGGGCGGCTCGCTGCTGCCCCCTAGCGGGGGAGGGACGTAATTACATCCCTGGGGGCTTTGGGGGGGGGCTGTCCCTCTAGAGCCCCATCCTCACTGACTCCGTCCTGGAGTTGGATGAGAGATAATGGCCTTACGTTGTGCCAGGGGAGGGTCGGGCTGGATTTAGCAAGATTTACCTTCTCCAAAGAGCGGTGCTGCAGTGGCACAGCTGCCCACGGAGGTGGGGGGGTCACCGTCCCTGGAGGTGATGAAGAACTGTGGGGATGTGGCACTGAGGGACATGGCCAGTGGGCACGGTGGGTGGGTTGGGGTTGGTCTTGGGGATCTTGGAGGGCTTTTCCAGCCTTCATGATTTGACGATTGTATGAACATCTACATGGCAATTCTCCAGCTGCCTGTCCCAGTCCTACTGACCCAGCTGTATCTCTCCAGGCAAGCTCTTCCACCCCTTCTGCTTGCATCCAGACACCATCAAACATGCAGGCTCAGACACAGGGACCAGCAGTGTCTGTGGCCTTTTTGTGCTCCTCTCCATGCTGGGTTTTAACTTGCTCTTTGTCCTTCTATCCTATCTTCTTATCCTTAAGGCTGTTCTGAACGCTGTGACTTGGAGAGTGTCCCAGAGCCCTCAACACCTGCATGTCCCACGTCCATGCTGTCCTGCACTTCCTTATCCCCAAGATCTGCCTCTCCGTGATGCACTGAATTGGCAAACATGTGTCACCCCAGACCAACAATGTCACAGCAAACTCCCCCTTGATAGGACAAGGGGGAATGGCTTTACACTGAGACAGGGGAGGTTTGGGTTGGATATGAGGAGGCAGTTTTTCCCCCAGAGGGTGGTGACGCACTGAACAGGTTGCCCAAGGAGGCTGTGGATGCCCCATCCCTGCAGGCATTCAAGGCCAGGCTGGATGTGGCTCTGGGCAGCCTGGGCTGCTGGTTGATGACCCTGCACATAGCAGGGGGTTGGATCTGGATGAGCACTGTGCTCCTTTGCAACCCAGGCCGTTCTATGATTCTGTCATTCTAAATCTCTCTTTCAGCCTAAAGCTTTTTCCCCGTATCCCCCCAGGTGTCTGCAGGCTCAAAGAGCAGCGAGAAGCGTTCAGAGGAAAGCGATCCCGTGCCACCTTCCCCGTGCCCGGGCTGTCCCCGCACGCTGCCGGCTCGGGGATGCGGGGGGAGCGCCGGACCGGAGCGGAGCCCCGGGCGGCTCGCTGCTGCCCCCTAGCGGGGGAGGGACGTAATTACATCCCTGGGGGCTTTGGGGGGGGGCTGTCCC

**Supplementary Table S6. Sequences of pUC57-sgRNA and pU6-*BsmBI*-UbC-LSL-Cas9.**

| Primer | Sequence |
| --- | --- |
| EGFP-t7-for | TGAGCAAGGGCGAGGAGCTGTTC |
| EGFP-t7-rev | CCATGTGATCGCGCTTCTCGTTG |
|  |  |
| *c-Maf*-t7-for | GGATGGCTTCAGAACTGGCAATG |
| *c-Maf*-t7-rev | ATCTCCTGCTTGAGGTGGTCTAC |
|  |  |
| *MafB*-t7-for | TGAGCCTCGCTTTTAGCGATGGC |
| *MafB*-t7-rev | CGAGTTTCTCGCACTTGACCTTG |

**Supplementary Table S7. Primers of PCR for T7EN1 and sub-cloning of EGFP, *c-Maf* and *MafB* segments.**
